# Childhood memories of food and eating in lower-income families in the United States: a qualitative study

**DOI:** 10.1186/s12889-021-10533-1

**Published:** 2021-03-24

**Authors:** Nicklas Neuman, Karin Eli, Paulina Nowicka

**Affiliations:** 1 Department of Food Studies, Nutrition and Dietetics, Uppsala University, SE-751 22 Uppsala, Sweden; 2 Warwick Medical School, University of Warwick, Coventry, UK; 3 Unit for Biocultural Variation and Obesity, School of Anthropology and Museum Ethnography, University of Oxford, Oxford, UK

**Keywords:** Commensality, Family meals, Poverty, Social cohesion, Socioeconomic status

## Abstract

**Background:**

Childhood obesity prevention initiatives emphasize healthy eating within the family. However, family-focused initiatives may not benefit children whose families lack economic and/or social resources for home cooking and shared meals. The aim of this paper is to examine how adults talk about and make sense of childhood memories of food and eating, with particular attention to understandings of family life and socioeconomic conditions.

**Methods:**

Semi-structured interviews with 49 adults in 16 families (22 parents and 27 grandparents of young children) were conducted in Oregon, United States. Most participants had experienced socioeconomically disadvantaged childhoods. The interviews were analyzed using thematic analysis, with a focus on the participants’ memories of food provision, preparation, and consumption in their childhood homes.

**Results:**

Two main themes were developed: (1) “Food and cohesion”, with the subthemes “Care and nurturance” and “Virtue transmission through shared meals”, and (2) “Food and adversity”, with the subthemes “Lack and neglect” and “Restriction and dominance”. The first theme captures idealized notions of food in the family, with participants recounting memories of care, nurturance, and culinary pleasure. The second theme captures how participants’ recollections of neglectful or rigidly restrictive feeding, as well as food discipline tipping over into dominance, upend such idealized images. Notably, the participants alternately identified poverty as a source of lack and as an instigator of creative and caring, if not always nutritionally-ideal, feeding. Thus, they remembered food they deemed unhealthy as a symbol of both neglect and care, depending on the context in which it was provided.

**Conclusions:**

Childhood memories of food and eating may express both family cohesion and family adversity, and are deeply affected by experiences of socioeconomic disadvantage. The connection between memories of food the participants deemed unhealthy and memories of care suggests that, in the context of socioeconomic disadvantage, unhealthy feeding and eating may become a form of caregiving, with nutrition considered only one aspect of well-being. This has implications for public health initiatives directed at lower-income families.

**Supplementary Information:**

The online version contains supplementary material available at 10.1186/s12889-021-10533-1.

## Background

Public health initiatives concerning young children’s diets and the prevention of childhood obesity usually focus on the family context [1, 2]. This is based on assumptions about the family as the main provider of food, parents’ function as role models and children’s food socialization at the dining table. Evidence supports family-based interventions [2], and family meal frequency is associated with beneficial psychosocial outcomes in youth [3] and nutritional health in children [4], although causality remains unclear. More broadly, from a social perspective, food and commensality (shared meals) have been identified as central in the regulation of eating, and, by extension, social communion and order [5, 6]. In families and other constellations, social eating is also linked with self-reported pleasure and joy [7–11].

However, eating in the family does not always have positive consequences. Anthropologist Richard Wilk has critiqued the gap between the ideology and the reality of the family meal [12]. In contrast to claims about family meals’ universal benefits, Wilk presents ethnographic documentation (his own and other researchers’) where the meal fostered conflict, dominance, shame and guilt, both among adults and in adults’ relationships to children [12]. Moreover, as other studies have shown, socioeconomic disadvantage may amplify the family meal as a site of potential adversity, given the competing demands of public health guidelines and families’ everyday lives. Low-wage jobs, food insecurity and social class hierarchies have been shown to impact on families’ food and eating patterns, and on feelings surrounding feeding and eating [13–16]. This suggests that, while lower-income parents seem quite aware of what constitutes nutritious feeding and idealized eating, they often experience difficulties in bridging between nutritional awareness and feeding practices. For example, in a recently published ethnography of low-income mothers in the United States, the “failure” of not having family meals was cited as a source of conflict, guilt, and shame in food insecure families [17]. For these mothers, tackling hunger while feeding their children healthily was an ongoing hardship, exacerbated by limited possibilities to provide meals that were nutritious, filling and affordable [18]. In another study from the United States, children and adolescents (12–19 years) of differing socioeconomic status spoke of unhealthy eating as physiologically negative and as a symbol of moral inferiority [19]. While adolescents of low socioeconomic status (SES) subscribed to these moralist views of food, their families could not practice healthy eating due to financial constraints, leading to feelings of embarrassment, shame, and moral failure [19]. Research from Australia has also attended to children’s experiences of food insecurity, demonstrating that children felt that hunger “marked” their bodies as vulnerable and “shameful”, and that families’ navigations of food insecurity often led to a co-existence of childhood obesity and hunger [20].

Although the literature sheds light on children’s and parents’ cross-sectional experiences of food insecurity, it remains unclear how adults remember experiences of food in their childhood homes, and whether these memories, with their potential for longitudinal implications, could enhance debates within public health and food policy. Therefore, in this paper, we ask: how do adults talk about and make sense of childhood memories of food and eating? Our aim is to examine this, with particular attention to participants’ understandings of how their childhood food experiences relate to family life and socioeconomic conditions. We explore memories of food through narratives told by adults of two generations, most of whom experienced socioeconomic hardship. By focusing on two generations of participants from lower-income families, our paper contributes an inter-generational dimension to the literature on the social stratification of food and eating.

## Method

The study was conducted in Eugene, Oregon, United States, in 2011. Forty-nine members (22 parents and 27 grandparents) of 16 low-income families were recruited through purposive sampling to participate in semi-structured interviews. The sample size was judged as satisfactory to reach data saturation [21]. As the study focused on parental and grandparental involvement in young children’s eating and physical activity, families of children age 3–5 years with a minimum of one parent and one actively involved grandparent were included. “Actively involved” grandparenting was defined as spending time with the child on at least two occasions each month. Since family constellations were not defined beforehand but rather based on participants’ self-reported involvement in the child’s life, the number of interviewed people per family ranged from two (one parent and one grandparent) to six (two parents, two grandparents and two step-grandparents).

To reach low-income participants, participants were recruited through advertisements in Craigslist and the jobseekers section of a local newspaper. Participants contacted the research team via phone or email. Each participant completed a sociodemographic questionnaire (Supplementary file 1) and took part in a one-on-one interview (lasting about 1.5–2.5 h). There were no repeat interviews and no other people were present at the interview occasion. The questionnaire included questions about education, employment, and family and living conditions (see Table 1). Moreover, anthropometric measures were taken (of both the interviewed adult and the child in focus), but these are not reported in the present paper. As the study utilized a mixed survey and semi-structured interview design, fieldnotes were not taken. Two female interviewers conducted the interviews, based on two pilot-tested interview guides (for parents and grandparents respectively) that were developed for this study and focused on the same main topics and questions (Supplementary file 2, Supplementary file 3). The first interviewer was the last author, a postdoctoral researcher with extensive experience and training in qualitative interviewing, and the second interviewer was a research assistant (with a bachelor degree in psychology), with experience in working with preschool-aged children.

**Table 1:** Sociodemographic details of participants. Adapted from Neuman et al. [22]

	Parent (*n* = 22)	Grandparent (*n* = 27)
**Mean age (range)**	32.2 (22.7–49.5)	56.9 (43.0–77.9)
**Sex:**		
Female	14 (64%)	21 (78%)
Male	8 (36%)	6 (22%)
**Race / ethnicity**		
Euro-American	20 (91%)	23 (84%)
Native American	1 (4.5%)	0
Asian American	1 (4.5%)	1 (4%)
African-American	0	1 (4%)
Mixed	0	2 (8%)
**Highest school grade completed**		
High school	18 (82%)	20 (74%)
College/University	4 (18%)	7 (26%)
**Marital status**		
Married	6 (27%)	10 (37%)
Separated	1 (4.5%)	1 (4%)
Divorced	7 (32%)	14 (51%)
Single (never married)	7 (32%)	1 (4%)
Engaged	1 (4.5%)	0
Widowed	0	1 (4%)
**Employment status**		
Full time	7 (32%)	8 (30%)
Part time	4 (18%)	4 (15%)
Not employed	11 (50%)	15 (55%)
**Annual household income**		
Less than 14.999 USD	8 (36%)	7 (26%)
15.000–24.999 USD	6 (27%)	6 (22%)
25.000–39.999 USD	4 (18%)	6 (22%)
More than 40.000 USD	4 (18%)	8 (30%)

In addition to questions that focused on the child, the interview guides included questions about the participants’ childhood memories of food and eating. We did not assume that participants’ responses reflected what had actually occurred. Rather, we treated their responses as reconstructions of life events that, in the context of the interview, participants expressed as meaningful. As demonstrated in previous research, food and drink can bring forth remembrances relevant to social identity, culture and tradition, as well as illustrate broader social transformation [23]. In other words, food memories can stimulate what C. Wright Mills saw as a fundemental aspect in the sociological imagination: connecting particular individual biographies into the broad context of history [24].

Interviews were video recorded and transcribed verbatim by students at the last author’s university. Transcriptions were not shared with the participants. The study was reviewed and approved by the Institutional Review Board at the Oregon Social Learning Centre, where the last author was a postdoctoral researcher and where all the interviews took place. Participants received an information sheet about the study, where they learned that the interviewers were interested in child development and family dynamics. All participants provided written informed consent. No potential participant who had been approached declined participation and the study had no drop-outs. Each participant was given 50 USD for their participation. Further details about the study’s methodology have been included in previous publications [22, 25–27].

### Analysis

Data were analyzed using thematic analysis [28]. The initial phases of thematic analysis involve the organization of data and familiarization with these data. In this case, all three authors – the second and last in particular – were already well acquainted with the data. There was, however, a need for reorganization based on the aim of this analysis. The first author, who led the analysis, systematically screened the transcripts for excerpts focused on memories of food. These were copied into a new document in which the first author began a theory-driven coding procedure, using a word processor and spreadsheet software. The preliminary codes were based on propositions derived from the literature on food, families and commensality. Initially, the analysis was narrowly focused on meals. However, having carefully reviewed and discussed the data, all authors agreed that such a delimited analysis would miss important details, since the meal as a particular occasion was analytically indistinguishable from broader stories about food in the family.

The first author coded the excerpts, focusing primarily on whether or not memories were framed as positive, negative or neutral and how food activities (eating, working with food etc.) were described in terms signaling social communion, discipline or “just food” (a code signifying that the participant talked about food in an indifferent manner). The coding was then reviewed by the other authors who modified the codes and made additions, and all three deliberated about the interim analysis.

**Fig. 1:**
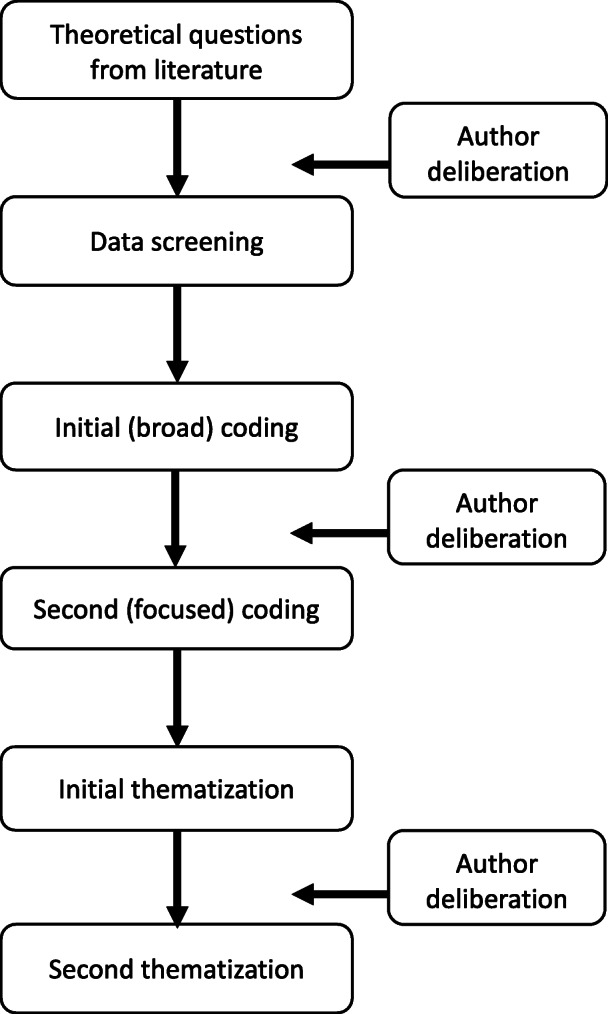
Flow chart of the analysis process

In the second stage of coding, the analysis focused on refining and adding to the initial codes. For example, additional codes were named “transmission of values”, “neglect”, “good care”, “bad care”, “good food”, “bad food”, “eat up” (when participants spoke about having to eat everything that was served), and “just the way it was” (e.g. a statement about what one used to eat or how meals were structured). The third stage of the analysis focused on abstracting the codes into themes. This was followed by further deliberation, including discussions about thematic saturation (when further analyses are judged redundant for the development of the theme). A second phase of thematization then took place, and two main themes with two respective subthemes were identified (for an illustration of the analysis process, see Fig. 1). These are presented below, with illustrative quotes. Findings were not discussed with participants. All participant names are pseudonyms and identifying details have been concealed.

## Results

The main themes we identified were “Food and cohesion” and “Food and adversity”. The first theme is divided into the subthemes “Care and nurturance” and “Virtue transmission through shared meals”. The second theme is divided into the subthemes “Lack and neglect” and “Restriction and dominance”.

### Food and cohesion

Many participants connected memories of food and eating in childhood to care and nurturance, encompassing both food itself and its association with affectionate relationships. In these memories, family meals and eating at home were cited as foundations of desirable values, food habits and behaviors. These positive memories framed food as an emblem of family cohesion, even when life was otherwise very hard.

#### Care and nurturance

When participants were asked about food memories in childhood, for example about their favorite foods or about the influence their grandparents had on their childhood diets, they often gave responses that went far beyond food and eating habits. Barbara, grandmother of 5-year-old Connor, described her relatives and family traditions when she spoke about food. “My father was always very warm and loving”, she said, adding that she “did have a very loving family unit, and family was really important. We went to church every Sunday, a lot of family gatherings, and about food too”. The interviewer went on to ask whether she remembered what she used to eat and drink, to which she said: “Oh, of course! We had specific things each holiday we had. Thanksgiving was the normal Thanksgiving, but you know like Christmas we’d have prime rib, and on Sundays my dad would cook breakfast, birthday cakes.” Thus, Barbara connected food with familial relationships and with family practices rooted in United States and Christian traditions, and she also explained how she was disciplined into eating vegetables even if she did not like them. Russell, grandfather of 5-year-old Ethan, expressed similar memories about the educating role that food played in his childhood.

As a youngster, we lived close enough to school, so for lunch we came home from school. My mother would have soup, or peanut butter sandwich, or grilled cheese. She’d have lunch waiting for us when we got home from school. Then usually in the afternoon, I’d come home hungry and have a couple bowls of cereal. At 5:30 or 6 dinner was always a family sit down. My dad would be home and we would always sit down as a family.

[ … ]

My mom cooked fairly well. We had steak, that was kind of a big thing if we had steak. If you weren’t home on time, if everyone finished their meal … there wasn’t any guarantee that your steak wasn’t already going to be gone. So it was kind of a punishment to not get home when you’re supposed to.

The quotes suggest the meal as a standard family gathering as well as an arena for discipline. Neither Barbara nor Russell described the disciplinary aspect of the meal as unfair, however, but as a way of bringing up children within a cohesive family unit. Similarly, Linda, grandmother of 3-year-old Edgar, mentioned that, as a child, she “didn’t like animal meat”, except “Kentucky Fried Chicken”. She further explained there was a rule in her childhood home “that you had to eat whatever was on your plate”, leading to potential difficulties when meat was served. However, Linda’s mother gave her small pieces of meat so that Linda “didn’t get into trouble with [her] dad”. In Linda’s story, meals were venues for discipline, but also revealed her mother’s flexible and caring feeding.

Several participants considered the family an important unit for fostering healthy dietary habits. In a few cases, perhaps counterintuitively, this was expressed as a byproduct of poverty. Many participants described growing up in poor farming families and having no choice but to eat fresh foods from their gardens. Patty, grandmother of 4-year old Kyra, came from a family of poor migrant farmers and said she ate healthily as a child “because we couldn’t afford the other food in the stores”. Therefore, Patty’s family “ate a lot of mashed potatoes, pinto beans … fried potatoes, biscuits and gravy. And then when we did fruit and stuff, we would eat the fruit, and my mom would can it”. While Patty talked at length about the hardships of poverty and how the family was hurt by abusive behavior and addiction, food still seemed to elicit good memories of healthy familial practices within an otherwise difficult and distressing home life.

Another side of food and care was exemplified in the story of Eve, mother of 5-year-old Kelvin. On the one hand, Eve recalled “really well balanced meals that we ate together”, but, on the other hand, she also remembered how

we always had junk food at home so it was never like I had to go out and go get it or anything like that, or sneak or anything. Just if I wanted it, it was there and if I didn’t it wasn’t ever an issue.

Eve had positive memories of food in her childhood home. Meals were “well balanced” and eaten within the family unit, but there was also individual access to treats, which she seemed to consider a good thing. The addition about her never having to “sneak or anything” suggests a notion of “junk food” consumption as an otherwise normatively regulated vice, although this was not the case in her family. This exemplifies that meaningful food memories of familial care and nurturance can center both on food considered healthy as well as on “junk food” and the freedom to snack individually.

#### Virtue transmission through shared meals

As hinted in some of the quotes above, participants described values and proper behavior – for example, what to eat or how to act – as disciplinary benefits of shared family meals. For example, Jane, mother of 5-year-old Kate, said that her family “always sit[s] down … and eat[s] dinner”. “[T]hat’s the best time to sit and talk too. It’s social”, she said, relating this contemporary routine to “how I was raised and that’s how the kids are going to be”. Her partner Sam (Kate’s father) shared the same sentiment. When asked about similarities between how he raised his daughter compared to how he was raised, he mentioned “the way, especially at mealtime, the value of the family”. He then continued:

Just carrying on the morals and the values instead of just creating your own. Both Jane and I have carried those same morals and values ‘cause we were both raised with very good morals and values, at least I think, otherwise we’d both be out killing and robbing.

Such conceptualizations of food and shared meals as devices for intergenerational connection were common, although expressed in varied ways. Jane mentioned how, as a child, she loved McDonald’s meals, especially chicken nuggets, a food that seemed central to her relationship with her father.

Chicken McNuggets … was a special thing and that’s how [my father] won my heart umm, yeah I don’t remember a whole bunch. I know that like everything was out of a box growing up. It’s like, it was never homemade. I just remember like, when I was learning how to cook everything like, Hamburger Helper, out of a box.

While food and mealtimes connected to positive memories of Jane’s family upbringing, hers was not a romanticized picture of particularly healthy food or idealized home cooking: the food shared was prepackaged and heavily processed, and Jane’s parents transmitted the knowledge of cooking “out of a box”. Similar to Eve’s story, described under the previous subthemes, Jane’s story exemplifies a complex association between care and feeding, in which food that might be labelled unhealthy is nonetheless associated with family cohesion.

Even when food memories were associated with impoverished or otherwise difficult life conditions, shared meals were often remembered positively. When Molly, grandmother of 5-year-old Kelly, spoke about her childhood memories of eating, these were imbued with disorder and idiosyncrasy, which she attributed to her parents’ relationship breakdown and her mother’s chronic illness. As an adult, despite living through much economic hardship, Molly valued the ordered family meals she did not have as a child. She had an ambivalent relationship to her being employed, since this meant a better financial situation but less time to cook and eat as a family. “[W]e were very poor but we did have decent food”, she said, adding that people around her might have thought otherwise since her family’s diet was low on meat and high in vegetables, something that “now I think it would be considered healthier”. She also mentioned having a

big dining room with a big table and all my kids sat at it together and I think things were okay then. It changed when I went to work, but I try to do that with my youngest one now and with Kelly when she’s there, because I would like her to have those memories.

The quote suggests a valuation of the shared family meal around a table, although this had become more and more problematic as Molly “worked [her] way up to the [manager’s] position which paid a lot more but took a lot more time”. And since this ideal was difficult to fulfil throughout her eldest children’s childhood, she now wanted to transmit this to her youngest child and her granddaughter, “because I would like her to have those memories”. As such, while Molly described food and meals as closely connected to ideals of family cohesion, these ideals had to be weighed against economic necessity. Her story therefore captured a gap between values and the ability to enact them, implicitly challenging family mealtime “prescriptions” whilst endorsing the assumptions that underlie them.

### Food and adversity

While memories of food and eating in childhood were often positive, this was not the case for all participants. Indeed, participants also spoke of food and eating in their childhood homes as associated with adverse experiences of lack, neglect, rigid dietary restriction, and parental dominance.

#### Lack and neglect

Given food’s close association with caregiving and well-functioning families, it is no surprise that memories of food and eating also capture the opposite. One example is Diane, step-grandmother of 3-year-old Seth, for whom the lack of mealtime sociality in childhood encapsulated a general lack of parental involvement. She said:

My dad worked all the time at the factory. … He would get off really early, at 3. But he would sit home and read the newspaper. And my mom, she wasn’t involved either. They were very much the, I guess just the family where the kids raise themselves. You sit down at the table and eat together but my brothers and sisters and I, we all talk about it.

When Diane said “it”, she referred to racial tensions at her school that neither she nor her siblings talked about with their parents. “I would drink nothing before I go to school”, she explained “[s]o that way I wouldn’t go to the bathroom. I wouldn’t get beat up.” But she kept it to herself. She continued, describing how “[m]y parents never knew anything going on with our school … they just kind of ran the house. My dad would work and read the newspaper, would sit and eat dinner together and go to bed.” Diane’s father’s working schedule and the absence of any meaningful interaction at the table exemplified lacking parental involvement and neglect toward his children’s life conditions. This seems to have affected Diane’s own parenting, with an imperative to be more involved and talk to her children: “I talk to my kids. My younger daughter talks to me a lot about what’s going on.”

In the previous section, we related Molly’s story of how family meals declined when economic need drove her to work more hours outside the home. David, Molly’s son, told the story from his perspective, detailing negative experiences at which Molly hinted. “So there was less family time and stuff like that”, he began, “and as far as eating habits, I think my mom had less time and she had a lot of kids at the time.” When his mother worked, David stayed at home with her partner at the time (not his father), who, according to Molly, had a drinking problem. “[H]e was a horrible influence as a parent”, David said:

I took nothing from him that was worth anything, I don’t think. But as far as eating habits, there was more canned vegetables, Hamburger Helper and more store-bought, quick prepared foods that didn’t provide as good as nutrients and is not as healthy. I try to live a much better lifestyle now that I have more choices.

The “canned vegetables, Hamburger Helper and more store-bought, quick prepared foods” illustrated a lack of parenting abilities and neglectful behavior, materialized through ultra-processed foods. David said he learned nothing from his stepfather in terms of food, and argued that the neglectful feeding he experienced inspired him to do the opposite as an adult.

Another example of low-quality diets being associated with neglectful parenting appeared in Bell’s story. Bell, 3-year-old Seth’s mother, grew up in a poor family with parents who emigrated from East Asia. When asked about what she used to eat, she recalled her childhood hardships:

We were really poor. So my parents weren’t around a lot. So I ate a lot of junk food. I don’t have any directions as far as food goes. What I should eat, what I shouldn’t eat. It was just like whatever was around the house or in the neighborhood. … I would go get junk food every day. When we sit down together for dinner together, but, aside from that, my mom didn’t really … she would cook and like “eat it or not”. So we didn’t really have structure. So we are just like really, really skinny. And people kind of make fun of me being so skinny. So that wasn’t cool. But yeah my eating habits were terrible and didn’t have much supervision because my parents weren’t around much.

In the previous section, we showed how “junk food” and snacks could be associated with caring relationships, but Bell’s story starkly contrasted with this. Elsewhere in her interview, Bell talked about how she was “[a]lways underweight” and “like anemic when [she] was younger”. Her story included more examples of neglect, often connected to eating and health and primarily focused on Bell and her sister, but also related to her parents’ self-care, or lack thereof. Notably, while other participants described being told to “clean their plates” as a form of nurturing discipline, Bell’s mother’s ambivalent “eat it or not” attitude exemplified how the family “didn’t really have structure”. However, Bell was not entirely judgmental toward her parents. She thought her parents were not “completely up there sometimes”, but connected this to their life conditions in the United States, the crisis of migration, and their background as poor farmers in their country of origin. This ambivalence – on the one hand, feeling that one’s parents neglected both themselves and their children, yet, on the other hand, understanding the circumstances that underlay their neglect – differentiated Bell from some other participants with stories of neglectful behavior.

#### Restriction and dominance

While some participants remembered food-related discipline positively, other participants associated food-related discipline with adversity. These participants recollected events when food was rigidly restricted or became an instrument of dominance, such that food-related discipline compromised children’s well-being. The rigid restriction of food and eating, as opposed to a healthy setting of boundaries around food, was a form of parental control that participants described as oppressive and damaging. Barbara, who, in the previous theme, described a “loving family unit”, also talked about her mother having a disciplining side to her views on food, insisting on the children eating vegetables at every meal and not leaving the table until the food was eaten. Barbara did not characterize these rules as negative, but, later in the interview, recollected a more troubling form of discipline:

My mother was really concerned about weight and appearance. … She put me on a diet when I was 11, and I look at pictures, and I’m like, I always had a little belly. But it’s like, it was like a real diet. I remember going to the movie theater, and instead she would send me with a hardboiled egg. And I remember canned asparagus; it was like a low carbohydrate diet I think. And her telling me … I must have been six years old, she said, “Oh if only you could wear those cute clothes”. … That is one of the reasons I swore I would never, never say anything about my children’s weight. I would try and control it at home, but not say anything, because it has impacted me my whole life.^1^

Although Barbara felt that some food discipline was worth transmitting, putting a child on a restrictive diet was out of the question. In another example, Jackie, mother of 5-year-old Ethan, described similar memories, which she said resulted in an unhealthy relationship with food and her body, leading to an eating disorder. She said her father “always said stuff” about her weight:

He was the commenter. My mom told him I was getting boobs when I was 10, and he was like “Oh, that’s just fat.” Stuff like that, cause he’s really judgmental, …

[ … ]

… because I was a girl, he was really judgmental about [my body]. So he’s been commenting about my weight constantly. My whole life.

[ … ]

As long as I can remember. I feel like he’s the one who gave me all my body image issues. Seriously.^2^

Jackie’s father’s rigid restriction of her eating was expressed through body-focused comments. This left long-lasting marks (“I feel like he’s the one who gave me all my body image issues”), and Jackie thought that if her brother had been “chubby” instead of her, “it wouldn’t matter. But because I was a girl, he was really judgmental about it.” Jackie’s mother Thelma (Ethan’s grandmother) further illuminated Jackie’s memories. She described her husband’s character as “strong [and] stoic” and talked about his “definite ideas” about meals, saying he is “more ritualized about food” and “has more rules around food, most of it is discipline kinds of rules”. She also confirmed the remarks about Jackie’s body and reaffirmed her view of him as particularly disciplinarian: “I remember him saying ‘Does she really need a bra, or is that just fat’ … He’s not going to preach to you about weight, he’s going to get you out and run you on the track”.

Another form of rigid dietary restriction, driven by neglect and abuse, appeared in the story of Wayne, Seth’s father, whose food memories were embedded in family conflicts and alcoholism. His parents Sally and Lance, who were both interviewed, separated when Wayne was 2 years old, and he said that Lance was verbally abusive both to him and to his mother. Wayne was always hungry when staying at his father’s house, he said, “because we’d get two bucks for school lunch and school lunch was never enough”. Furthermore, his stepfather Doug (also interviewed) used to “talk a lot about how I ate when I was younger. It’s funny, I never turned into a fat kid. I have never been an emotional eater. But my stepdad gave me a really hard time about how I ate.” Wayne’s interview represents a complex case in which food memories were interwoven with memories of hunger, addiction, conflict, and poverty, upending over-generalized notions of how food brings families together to the benefit of children’s health and well-being.

## Discussion

Drawing on semi-structured interviews with 49 adults of two generations, most of whom had experienced socioeconomic hardship in childhood, this study found that memories of food and eating reflected both cohesion and adversity in family life. Cohesion was expressed through remembrances of care, love and culinary pleasure, with the family meal perceived as a source of virtue transmission, where values of connection, communication and good behavior were nurtured through generations. Adversity was expressed through remembrances of neglectful or deficient feeding and parenting, and through memories of damagingly restrictive discipline and dominance. Strikingly, experiences of poverty greatly imprinted on food memories, regardless of whether participants recalled joy or suffering.

Our finding that childhood memories of food and eating expressed family cohesion aligns with the situating of the family meal as a site for nutrition education and obesity prevention [1, 2]. However, our finding that memories of food and eating also expressed family adversity problematizes the idealization of the family meal. This finding aligns with critiques that position family meals as a source of conflict, shame and dominance [12, 17]. In our analysis, adversity related to memories of food and eating had two dimensions: neglectful parenting associated with deficient feeding, and abusive comments about children’s bodies and eating associated with rigidly restrictive feeding. The latter carry longstanding implications for body image and disordered eating, as we have described at length elsewhere [27]. These findings convey that the promotion of family-focused interventions into children’s eating should account for both the positive and the negative realities of food in family life. This is particularly important given that experiences of abuse in childhood (including emotional abuse) have emerged as a potential risk factor for obesity in adulthood [29].

A key finding was that participants remembered food they deemed unhealthy as a symbol of both care and neglect, depending on the context in which it was provided. The connection between care and what participants in our study called “junk food” suggests that, in the context of socioeconomic disadvantage, unhealthy feeding and eating may become a materialized form of care. This finding aligns with a previous study, based on 160 interviews and 80 h of observations with families in the United States, which linked the symbolic value of food with class-based notions of parenting [15]. Among low-SES families, food was used to compensate for other forms of scarcity, and food provision enabled parents to meet children’s emotional needs and reinforce their own worth as caregivers. In other words, when material resources were scarce, food considered unhealthy became a way for parents to treat their children [15]. This suggests that unhealthy eating cannot be reduced to a problem of knowledge, cost or access, but must also be understood as a form of caregiving, where nutrition may have to be weighed against other aspects of well-being. As previous studies have suggested, it is important to consider lower-income families’ food experiences and concepts of care when designing healthy eating interventions [30, 31]. Our findings reinforce this call.

This study has both strengths and limitations, primarily pertaining to the design and to the nature of the data. Interviewing several people in the same families provided us with rich data that, had we interviewed 49 people from 49 different families, we would not have obtained. The study design allowed us to triangulate participants’ life stories in ways that challenged, confirmed, and added complexity to their narratives. The sampling strategy also gave us insight into the dynamics of extended family relations beyond parents and children. However, in the present analysis, the study design could be seen both as a weakness and as a strength. Since the original research questions mainly focused on the participants’ preschool-aged children and grandchildren, interviews emphasized domains such as children’s dietary habits and physical activity. Thus, it is possible that a different study design would have allowed for further investigation of the participants’ memories of food and eating, leading to more complex findings. At the same time, the fact that many stories included in this analysis emerged spontaneously, despite not being part of the original interview design, is a testament to how much these food memories mattered. Another limitation is the ethnic homogeneity of the study sample. The great majority of participants was white, which may affect the findings’ applicability to other ethnic groups, even with comparable socioeconomic conditions.

Moreover, as mentioned in the method section, reliance on long-term memories is subject to considerable bias. We do not claim that participants’ food memories perfectly match reality or that causal inferences can be drawn from childhood experiences to the participants’ lives at the time of interview. However, because these experiences were meaningful to the participants, who cited them as underlying their contemporary values and daily activities, this suggests that memories of food and eating can tell us something substantial about peoples’ life worlds and the sociocultural environments in which they live [23]. Finally, external validity is always an issue for data collected through qualitative interviews. Interpretations that extend the study’s findings to other national or social contexts must therefore be done with caution, and potential transferability should be carefully considered. To strengthen transferability, we suggest that researchers who wish to apply our findings to other populations contextualize these within socioeconomic and demographic data regarding these populations.

## Conclusions

Childhood memories of food and eating may express both family cohesion and family adversity, and are deeply affected by experiences of socioeconomic disadvantage. The connection between memories of food the participants deemed unhealthy and memories of care suggests that, in the context of socioeconomic disadvantage, unhealthy feeding and eating may become a form of caregiving, with nutrition considered only one aspect of well-being. This has implications for public health initiatives directed at lower-income families.

## Supplementary Information

**Additional file 1.**

**Additional file 2.**

**Additional file 3.**

## Data Availability

The datasets used and analyzed during the current study are available from Paulina Nowicka, the project investigator, on reasonable request.

## Footnotes

1 This quote also appears, in a slightly different form, in a previous publication [27].

2 This quote also appears, in a slightly different form, in a previous publication [27].
