# Supplementary material for: Childhood memories of food and eating in lower-income families in the United States: a qualitative study
Source: BMC Public Health. 2021 Mar 24;21:586. doi: 10.1186/s12889-021-10533-1 (PMC7992930; doi:10.1186/s12889-021-10533-1)
Supplement: Supplementary file 1 — Additional file 1. [file 12889_2021_10533_MOESM1_ESM.doc]

## **DATE: __ __/__ __/__ __ __ __ STAFF: __ __**

**Demographic Questionnaire**

1. **Your** date of birth: ___ ___/___ ___/___ ___ ___ ___

Mo Day Year

2. What is your relationship to the child in the Grandparents Study ? Please circle one answer.

**(*If you have a biological relationship to the child or are related by marriage to the child, please select that answer over any other relationship such as foster, adoptive, etc. If unsure of which category to choose, please write us a note explaining your situation.)**

1 -bio mother 7 -foster mother

2 -bio father 8 -foster father

3 **-**step mother 9 -adoptive mom

4 -step father 10 -adoptive dad

5 -grandmother 11 -other relative (specify: ______________________________)

6 -grandfather 12 -other (specify: _____________________________________)

3. How many people live in your household including yourself? Total: ___ ___

4. What is your current legal marital status?

1 - Married

2 - Separated

3 - Divorced

4 - Single (never married)

5 - Widowed

6 - Other (explain:____________________________________________________ )

5. How many times have you been married? ______ times

6. Are you of Hispanic or Latino ethnicity? 1 - Yes 2 – No

7. Is your child of Hispanic or Latino ethnicity? 1 - Yes 2 - No

8. What is your racial category? Please circle all that apply.

1 - Native American/American Indian or Alaska Native

2 - Asian

3 - Native Hawaiian or Other Pacific Islander

4 - African American/Black

5 - Euro-American/Caucasian/White

6 - Other (specify: ______________________________ )

9. What is your child’s racial category? Please circle all that apply.

1 - Native American/American Indian or Alaska Native

2 - Asian

3 - Native Hawaiian or Other Pacific Islander

4 - African American/Black

5 - Euro-American/Caucasian/White

6 - Other (specify: ______________________________ )

10. Which number represents the highest grade you completed in school?

1 - Below 6^th^ grade

2 - Partial junior high (6^th^ or 7^th^)

3 - Junior high school (8^th^)

4 - Partial high school (9^th^-11^th^)

5- G.E.D

6 - High school (private, parochial, prep, trade or public)

7 - Some community college or vocational/specialized training courses

8 - Partial 4-year college (less than one year)

9 - Partial 4-year college (at least 1 year)

10 - Specialized/vocational training completed

11 - Community college degree or certification

12 - Standard 4-year college or university graduation

13 - Some graduate courses

14 - Graduate degree

11. Are you currently in any kind of college or training program?

1 - No

2 - 4-year college

3 - 2-year community college

4 - Vocational training (e.g. secretarial, cooking school)

5 - Other (specify: ________________________________ )

12. Are you currently working outside the home or at an in-home business?

1 - Full time

2 - Part time

3 - Not employed **Please skip to Question 13**

12a. How many hours a week do you currently do this work? __ __ hours a week

12b. What shift do you work?

1 - Day

2 - Swing

3 - Graveyard

4 - Variable Shifts

***If you have more than one job, please tell us only about your main job for Questions 12c~12g**

12c. What is your current occupation or your job title? _____________________________________

12d. What do you do in your job? What are your responsibilities?_______________________________

___________________________________________________________________________________ ___________________________________________________________________________________

___________________________________________________________________________________

12e. What kind of training was required or did you need for this position?________________________

___________________________________________________________________________________ ___________________________________________________________________________________

12f. Do you supervise anyone?

1 - Yes 🡪 If yes, how many people? ________

2 - No

12g. Are you a business owner/partner?

1 - Yes

If yes, how much is your business worth? $____________ **Please skip to Question 16**

2 - No **Please skip to Question 16**

13. Have you ever been employed?

1 - Yes

2 - No **Please skip to Question 14**

13a. What was the last job you had? Job title: ______________________________________________

13b. What did you do in your job? What were your responsibilities?____________________________

___________________________________________________________________________________ ___________________________________________________________________________________

___________________________________________________________________________________

13c. What kind of training was required or did you need for this position? ______________________

___________________________________________________________________________________ ___________________________________________________________________________________

13d. Did you supervise anyone?

1 - Yes 🡪 If yes, how many people? ________

2 - No

13e. How long have you been unemployed? ___ ___ years ___ ___ months

14. What is the main reason you are currently unemployed? (If more than one reason , please write the **primary or main** reason on the line below)

_________________________________________________________________________________

15. Are you currently looking for work?

1 - Yes

2 - No

16. Altogether, how many months have you worked for pay in the past 12 months? ___ ___ months

17**.** Which number represents your gross annual household income?

1 - Less than $4,999 7 - $30-39,999

2 - $5-9,999 8 - $40-49,999

3 - $10-14,999 9 - $50-59,999

4 - $15-19,999 10 - $60-79,999

5 - $20-24,999 11 - $80-99,999

6 - $25-29,999 12 - $100,000 +

18. Do you live in a… (please circle one)

1 - Single family home

2 - Mobile home

3 - Duplex

4 - Apartment

5 - Homeless

6 - Other (describe: _____________________________________________________ )

19. **In the past year**, has your family received any of the following types of assistance? Circle all that apply.

1 - No financial assistance

2 - Food stamps

3 - TANF (Temporary Assistance to Needy Families)

4 - Other welfare

5 - Medical

6 - Low income housing

7 - SSI (Supplemental Security Income)

8 - School loans and/or grants

9 - Unemployment insurance

10 - Other (describe: _____________________________________________________ )

20. Please think about how you feel about your family’s economic situation and indicate how much you would agree or disagree with each statement.

**Strongly Neutral/ Strongly**

**agree Agree Mixed Disagree Disagree**

My family has/We have enough money to afford the kind of…

a. home we would like to have 1 2 3 4 5

b. clothing we should have 1 2 3 4 5

c. furniture or household equipment we should have 1 2 3 4 5

d. car we need 1 2 3 4 5

e. medical care we should have 1 2 3 4 5

f. leisure and fun activities we want to participate in 1 2 3 4 5

21. Think back over the past year and tell us how much difficulty you had with paying your bills. Would you say you had…

1 - A great deal of difficulty

2 - Quite a bit of difficulty

3 - Some difficulty

4 - A little difficulty

5 - No difficulty at all

22. Think again over the past 12 months. Generally, at the end of each month do you end up with…

1 - Not enough money to make ends meet

2 - Just enough to make ends meet

3 - Some money left over

4 - More than enough money left over

**END - THANK YOU**
