# Supplementary material for: Childhood memories of food and eating in lower-income families in the United States: a qualitative study
Source: BMC Public Health. 2021 Mar 24;21:586. doi: 10.1186/s12889-021-10533-1 (PMC7992930; doi:10.1186/s12889-021-10533-1)
Supplement: Supplementary file 2 — Additional file 2. [file 12889_2021_10533_MOESM2_ESM.doc]

**Questions for parents**

**Introduction**

This interview is about the lifestyle of young children and the role of family. We understand that parents and grandparents influence what children eat and how much they play. We would like to learn more about your experiences and thoughts about the role of family members. We would also like to talk a little about your child’s weight, as we know it is an important issue for many families. We believe that there are no wrong or right answers. Your answers are most helpful in our work at the Oregon Social Learning Center on programs for families with preschool children.

**Opening questions**

Let us start out with some general questions.

1. How many children do you have? (Probe: In total, how old are they, where, and with whom, do they live. Note if the child is parent’s biological child. Draw a family tree).

**Your child and food**

1. Now I would like to focus on x (the child the interview is about). So, how would you describe x? (Quiet, bouncy?). From whom (which parent) did he/she get this from?
2. Is your child the type of child who likes all kinds of food or is he/she picky?
3. Is he/she usually hungry and likes to eat often or not hungry most of the time? And how do you deal with that (his/her appetite or lack of it)?
4. So what does your child like to drink?
5. And what does he/she drink most often? (Probe: How about water? Milk? Juice? Regular soda? Diet soda?).
6. What do you think about those choices? Good, bad? How come?
7. Do you try to influence his/her choice of drink?
8. What is your child’s favorite food? And least favorite food? (If you are not sure, could you guess?)
9. Many parents say that their kids put pressure on them to buy specific foods. Does it ever happen that your child asks you to buy that you are not sure you should? If so, how do you deal with these requests?

**Your child & the ways to be active**

Now I would like to ask you a few questions about the way your child spends his/her time and what you think about it.

1. What do you think about *how much* your child is playing/being active? (Probe: What about meeting friends, watching TV, playing video games, sitting in front of the computer, doing sports, having hobbies, other activities, going out with the dog, helping the family clean or do shopping…)
2. How do you think a child *should* spend his/her time? What activities are good for the child? (Probe: What about meeting friends, watching TV, playing video games, sitting in front of the computer, doing sports, having hobbies, other activities, going out with the dog, helping family with the household or groceries…)
3. How do you try to encourage your child to be more active?
4. Many parents usually say that their kids put pressure on them to be able to spend a lot of time watching TV and sitting in front of the computer. Does it ever happen to your child and if so, how do you deal with these requests? What has been most effective?

**Communication in the family**

1. Do you discuss what your child eats and drinks with your child’s grandparents? If yes, how, if not, why?
2. What do you do if you and your child’s grandparents don’t have the same opinion about how much and what your child should eat?
3. Overall, when it comes to food, do you think that the traditions differ a lot between your generation and when you were preschool age (that is your parents’ generation)?
4. Do you discuss your child’s activities (playing inside/outside, spending time in front of the tv/computer, meeting friends) with your child’s grandparents? (If yes, how? If no, why?)
5. What do you do if you and your child’s grandparents disagree about how your child spends his/her time?
6. Also, when it comes to spending time (=child’s physical activity), do you think that the traditions differ a lot between your generation (you are parent) and when you were preschool age (that is your parent’s generation)?
7. In general, how do you view your role on your child’s lifestyle? (Probe: What is most challenging? Out of the behavior management techniques you use on your child, which do you see as especially effective in influencing food/play?)
8. How do you think the grandparents of your child view your role on your child’s eating and activities? (Probe: What do you think your child’s grandparents recognize about the way you influence your child positively?
9. And how do you view grandparents’ role in your child’s lifestyle? (Probe: Out of the behavior management techniques they use on your child, which do you see as especially effective in influencing food/play?)

**Weight**

Now I would like to ask you a few weight-related questions.

1. Do you think that how much a child weighs matters? If yes, why? If not, why?
2. How much do you think that a child’s weight is possible to control/controllable? (=possible to change by changing the lifestyle)
3. *If yes,* what lifestyle habits do you think are the most important? How/when do you think they can be promoted, and who do you think can do that? And who in the family plays the most important role when it comes to influencing the child’s weight?

*If no,* what makes you think that way?

1. What do you think about your child’s weight? (Probe: As compared to his/her siblings, cousins, other children. Are you concerned/not concerned?)
2. What do you think that the grandparents of your child think about your child’s weight? (Examine: If there is more than one grandparent, do they have the same opinion?)
3. Do you talk about your child’s weight with his/her grandparents? (If yes, why, how? If not, why? Examine: If there is more than one grandparent, which of them do you talk the most with and why?)
4. Do you know if your child thinks about his/her weight? (Probe: Does he/she ever comment on it? Did that happen in your presence? If yes, what did you say? If your child doesn’t think about his/her weight, is it good or bad?)

**When you were a child**

Let’s go back in time… I wonder what you remember about your own childhood, especially your early childhood. We’ll focus mostly on your lifestyle habits, that is food and physical activity.

1. Do you remember what you used to eat and drink?
2. What was your favorite food? Could you eat that as much as you wanted?
3. What was your least favorite food? How often did you have to eat that food (you didn’t like)?
4. How did you spend your days when you were around 5-8 years old? (Probe: How much did you play with your siblings, friends, played inside/outside, helped with the household, did you have any hobbies?).
5. What was your favorite way of spending time when you were preschool/early school age? Did you have to negotiate that with your parents?
6. Did you think about your weight when you were a child, around 5-8 years of age? (If you compared yourself to your brothers and sisters, cousins, friends, other children).
7. At what age, if ever, did you start thinking about your weight?
8. What was your parents’ role in shaping your eating and drinking habits/physical activity? What did they do, what did they say? What do you remember the most? And if you had siblings, were your parents acting the same toward all children?
9. Provided that *both* parents were around: Were your parents equally involved in those issues? If not, which one of them was most involved?
10. Do you remember your grandparents; how did they influence you when it comes to these issues (food and physical activity)? If there is more than one grandparent, which of them was most involved?)
11. Do you remember if your parents and your grandparents agreed on these issues?

**Intervention issues**

We are almost at the end of our interview and I would like to ask you some last general questions, summing up your experience as a parent.

1. How can today’s parents create a good home environment with regard to food and drinks? And with regard to physical activity for their children? (Probe: How much do you think that the parents should influence children’s food choices/portion sizes? How much do you think that the parents should influence children’s time if front of TV/computer?)
2. What is most challenging for today’s parents?
3. What do you think may motivate parents to be more involved in shaping a good lifestyle at home?
4. In many places in the U.S. and overseas, researchers or health care providers are trying to organize programs that will help families with preschool children to develop good lifestyles and healthy weight in children. Do you think that such programs sound like a good idea? If yes, why? If not, why?
5. If yes, and if *you* were in charge of such a program for preschoolers, what would you do?
6. What would be the best way to let families in your community know about a program like this?
7. Lastly, do you think that such programs are more important to prevent unhealthy weight in children (that is it is better to involve families before children weigh too much) or do you think that it is better to wait and treat children when they weigh too much?
8. Is there anything else you would like to add or comment about?

*Thank you for sharing with your experiences*.
