# Supplementary material for: Childhood memories of food and eating in lower-income families in the United States: a qualitative study
Source: BMC Public Health. 2021 Mar 24;21:586. doi: 10.1186/s12889-021-10533-1 (PMC7992930; doi:10.1186/s12889-021-10533-1)
Supplement: Supplementary file 3 — Additional file 3. [file 12889_2021_10533_MOESM3_ESM.doc]

**Questions for grandparents**

**Introduction**

This interview is about the lifestyle of young children and the role of family. We understand that parents and grandparents influence what children eat and how much they play. We would like to learn more about your experiences and thoughts about the role of family members. We would also like to talk a little about child weight, as we know it is an important issue for many families. We believe that there are no wrong or right answers. Your answers are most helpful in our work at the Oregon Social Learning Center on programs for families with preschool children.

**Opening questions**

Let us start out with some general questions.

1. How many grandchildren do you have? (Probe: In total, how old are they, who is the parent, where do they live. May want to note if grandchild is biological. Draw a family tree).
2. How often do you see your grandchildren? (Probe: Each grandchild. Which of them do you see the most and why?).

**You and your grandchild**

1. Now I would like to focus on x (the grandchild the interview is about). How would you describe x? (Quiet, bouncy?). From whom (which parent) did he/she get this from?
2. What is your favorite way of spending time with your grandchild? How often do you spend time in that way?
3. What do you usually do otherwise? What do you do most often and why? Is there anything else you would like to do?
4. What or who influences the way you and the grandchild spend time together?

**Your grandchild & food**

1. I would like to ask you a few questions about what your grandchild usually eats and drinks and what you think about it.
2. Is your grandchild the type of child who likes all kind of food, or is he/she picky?
3. Is he/she usually hungry and likes to eat often, or not hungry most of the time? And how do you deal with that (his appetite or lack of it)?
4. So what does your grandchild like to drink?
5. And what does he/she drink most often? (Probe: How about water? Milk? Juice? Regular soda? Diet soda?).
6. What do you think about those choices? Good, bad? How come?
7. Do you try to influence their choice of drink?
8. Do you know what your grandchild’s favorite food is? And least favorite food? (If not, could you guess?)
9. Parents say that their kids put pressure on them to buy specific foods. Does it ever happen that your grandchild asks you to buy that you are not sure you should? If so, how do you deal with these requests?

**Your grandchild & the ways to be active**

1. Now I would like to ask you a few questions about the way your grandchild spends his/her time and what you think about it.
2. What do you think about *how much* your grandchild is playing/being active? (Probe: What about meeting friends, watching TV, playing video games, sitting in front of the computer, doing sports, having hobbies, other activities, going out with the dog, helping the family clean or do shopping…)
3. How do you think a child *should* spend his/her time? What activities are good for the child? (Probe: What about meeting friends, watching TV, playing video games, sitting in front of the computer, doing sports, having hobbies, other activities, going out with the dog, helping family with the household or groceries…)
4. How do you try to encourage your grandchild to be more active?
5. Parents usually say that their kids put pressure on them to be able to spend a lot of time watching TV and sitting in front of the computer. How do you deal (or would deal) with these requests? What has been most effective?

**Communication in the family**

1. Do you discuss what your grandchild eats and drinks with the child’s parents? If yes, how, if not, why?
2. What do you do if you and the child’s parents don’t have the same opinion about how much and what your grandchild should eat?
3. Overall, when it comes to food, do you think that the traditions differ a lot between when your children were preschool age (your generation) and now, when your child’s children are preschool age (your child’s generation)?
4. Do you discuss your grandchild’s activities (playing inside/outside, spending time in front of the tv/computer, meeting friends) with the child’s parents)? (If yes, how? If no, why?)
5. What do you do if you and your grandchild’s parents disagree about how your grandchild spends his/her time?
6. Also, when it comes to spending time (that is child’s physical activity), do you think that the traditions differ a lot between when your children were preschool age (your generation) and now, when your child’s children are preschool age (your child’s generation)?
7. Out of the behavior management techniques you use on your grandchild, which do you see as especially effective in your influence on your grandchild’s food/play?
8. In general, how do you view your role in your grandchild’s lifestyle when compared to the role of the parents?
9. How do you think the parents of your grandchild view your role in your grandchild’s eating and activities? (Focus: What do you think your grandchild’s parents recognize about the way you influence your grandchild positively?

**Weight**

1. Now I would like to ask you few weight related questions.
2. Do you think that how much a child weighs matters? If yes, why? If not, why?
3. How much do you think that a child’s weight is possible to control/controllable? (=possible to change by changing the lifestyle)
4. *If yes,* what lifestyle choices do you think are the most important? How/when do you think they can be promoted, and who do you think can do that? And who in the family plays the most important role when it comes to influencing the child’s weight?

*If no,* what makes you think that way?

1. What do you think about your grandchild’s weight? (Probe: As compared to his/her siblings, cousins, other children, to the child’s parents. Are you concerned/not concerned?)
2. What do you think that the parents of your grandchild think about your grandchild’s weight? (Examine: If there are two parents in the house, do they have the same opinion?)
3. Do you talk about your grandchild’s weight with his/her parents? (If yes, why, how? If not, why? Examine: If there are two parents in the house, which of them do you talk the most with and why?)
4. Do you know if your grandchild thinks about his/her weight? (Probe: Does he/she ever comment on it? Did that happen in your presence? If yes, what did you say? If your child doesn’t think about his/her weight, is it good or bad?)

**Now and then**

1. What do you think about how preschool children nowadays eat and drink?
2. What do you think about how preschool children nowadays spend their time (Probe: How much do they play with brothers & sisters/friends, sit in the front of the computer/TV, play outdoors/indoors?

**When you were a parent**

1. Even if it was quite a long time ago, I wonder what you remember about how things were at your home when you were a parent and your children were preschool age… So I wonder, what did your children usually eat?
2. Did you eat out as a family? If yes, how often?
3. Did you cook? Did you like to cook? Did you have time to cook?
4. Do you remember what your children use to drink? (Probe: How about water, juice, milk, sodas, chocolate milk?)
5. Did you as a parent have to negotiate eating with your children? Did you discuss/control how much, what and when they should eat?
6. How did your children spend their time when they were preschool age? (Probe: How much did they meet friends, played inside/outside, watched TV, helped with household, had hobbies).
7. What were your children’s favorite activities? What did *you* like the most that your children did? And how did you encourage that?
8. In general, how did you see your role in shaping your children’s lifestyle? What was most difficult? What was most effective?
9. If you had more than one child, did you have the same rules toward all children?
10. Was there another adult in the household… and if yes, did that person have similar ideas about what the children should eat and how much they should be active? If no, why?
11. What did you think about your children’s weight when they were preschool age? (Compared to other children. Were you concerned/not concerned? If you were concerned, did you do anything special about it?)
12. Did your children’s weight change a lot when they got older? During adolescence?
13. In general, did you think it was easier or more difficult to be a parent at those times? What was easier? What was more difficult?

Would you do anything different with the knowledge and experience you have now? If yes, what?

**When you were a child**

1. Now we are going even further back in time, so I wonder what you remember about your own childhood, especially your early childhood. How would you describe yourself as a child? (Quiet, bouncy?) From whom do you think you got this temperament?
2. Do you remember what you used to eat and drink?
3. What was your favorite food? Could you eat that as much as you wanted?
4. What was your least favorite food? How often did you have to eat that food (you didn’t like)?
5. How did you spend your days when you were around 5-8 years old? (Probe: How much did you play with your siblings, friends, played inside/outside, helped with the household, did you have any hobbies?).
6. What was your favorite way of spending time when you were preschool/early school age? Did you have to negotiate that with your parents?
7. Did you think about your weight when you were a child, around 5-8 years of age? (If you compared yourself to your brothers and sisters, cousins, friends, other children).
8. At what age, if ever, did you start thinking about your weight?
9. How was your parents’ role in shaping your eating and drinking habits/physical activity? What did they do, what did they say? What do you remember the most? And if you had siblings, were your parents acting the same toward all children?
10. Provided that *both* parents were around: Were your parents equally involved in those issues? If not, which one of them was most involved?
11. Do you remember your grandparents; how did they influence you when it comes to these issues (food and physical activity?)
12. Do you remember if your parents and your grandparents agreed on these issues?

**Intervention issues**

We are almost at the end of our interview and I would like to ask you some last general questions, summing up your experience as a parent and a grandparent.

1. How can today’s parents create a good home environment with regard to food and drinks? And with regard to physical activity for their children? (Probe: How much do you think that the parents should influence children’s food choices/portion sizes? How much do you think that the parents should influence children’s time if front of TV/computer?)
2. What is most challenging for today’s parents with preschool children?
3. What do you think may motivate parents to be more involved in shaping a good lifestyle at home?
4. In many places, in the U.S. and overseas, researchers or health care providers are trying to organize programs that would help families to develop good lifestyles and healthy weight in children. Do you think that such programs sounds like a good idea? If yes, why? If not, why?
5. If yes, and if *you* were in charge of such a program for preschoolers, what would you do?
6. What would be the best way to let families in your community know about a program like this?
7. Lastly, do you think that such programs are more important to prevent unhealthy weight in children (that is it is better to involve families before children weigh too much) or do you think that it is better to wait and target children when they weigh too much?
8. Is there anything else you would like to add or comment about?

*Thank you for sharing with your experiences*.
